# Associations between depression, domain-specific physical activity, and BMI among US adults: NHANES 2011-2014 cross-sectional data

**DOI:** 10.1186/s12889-022-14037-4

**Published:** 2022-08-25

**Authors:** Emily R. Rutherford, Corneel Vandelanotte, Janine Chapman, Quyen G. To

**Affiliations:** Central Queensland University, School of Health, Medical and Applied Sciences, Appleton Institute, Rockhampton, Australia

**Keywords:** Body mass index, Moderator, Mental Health, Exercise, Leisure, Occupational, Active transport

## Abstract

**Background:**

Physical activity is associated with depression. However, benefits of physical activity on depression may differ for specific domains of physical activity (i.e., leisure-time, work, and travel). Moreover, the relationship between physical activity and depression could also differ for people in different Body Mass Index (BMI) categories. This study investigated the relationship between domain-specific physical activity and BMI with depression, and the moderation effects of BMI on the relationship between domain physical activity and depression.

**Methods:**

Complex survey data from the NHANES 2011-2014 was used (N=10,047). Depression was measured using the Patient Health Questionnaire (PHQ-9). Participants reported physical activity minutes in each domain using the Global Physical Activity Questionnaire. Demographic characteristics were self-reported. Weight and height were objectively measured and used for calculating BMI. Survey procedures were used to account for complex survey design. As two survey cycles were used, sampling weights were re-calculated and used for analyses. Taylor series linearisation was chosen as a variance estimation method.

**Results:**

Participants who engaged in ≥150 minutes/week of total moderate-vigorous physical activity (MVPA) (adjusted B = 0.83, 95% CI [0.50, 1.16]) and leisure-time MVPA (adjusted B = 0.84, 95% CI [0.57, 1.11]) experienced lower levels of depression compared to those engaging in <150 MVPA minutes/week. Work and travel-related physical activity were not associated with depression. Overweight (adjusted B = -0.40, 95% CI [-0.76, -0.04]) and underweight/normal weight participants (adjusted B = -0.60, 95%CI [-0.96, -0.25]) experienced less depressive symptoms compared to obese participants. BMI did not moderate the relationship between domain-specific physical activity and depression.

**Conclusions:**

Interventions that focus on leisure-time physical activity appear to be best suited to improve depression, however, this needs to be confirmed in purposefully designed intervention studies. Future studies may also examine ways to improve the effectiveness of work and travel physical activity for reducing depression.

**Supplementary Information:**

The online version contains supplementary material available at 10.1186/s12889-022-14037-4.

## Introduction

Depression is a major contributor to the overall global burden of disease and is one of the leading causes of disability worldwide [1]. Depression, at its worst, can lead to suicide, with close to 800,000 people dying by suicide each year globally [1]. Depression often results from a mix of social, psychological and biological factors, with adverse life events, such as trauma or death of a loved one [1]. However, a systematic review found an increasing global trend of depression prevalence over time [2]. Studies also found prevalence of depression increased in the U.S. For example, data among adults aged >20 years showed an overall increasing trend of depression between 2005 and 2016 [3]. Another study used the National Survey on Drug Use and Health also found that prevalence of depression among people aged 12 and older in the U.S. increased between 2005 and 2015 [4].

The World Health Organization notes that physical health and depression are interrelated, and community approaches, such as physical activity programs, have been implemented to reduce depression [1]. The Royal Australian and New Zealand College of Psychiatrists clinical guidelines for mood disorders have also listed physical activity as a therapeutic option for depression [5]. Physical activity has similar effects as antidepressants [6] and psychotherapy [7]. A meta-meta analysis found that physical activity had a medium effect on reducing depression [8]. However, there is a lack of evidence for recommendations regarding duration, intensity, mode, or frequency of physical activity to understand what is needed for the anti-depressive benefits of physical activity to occur [9, 10]. It is currently recommended that people participate in at least 150 minutes a week of moderate-vigorous intensity activity (MVPA) for health benefits [11].

Physical activity can occur across many domains, including recreation or leisure-time (e.g., walking, organized or self-directed exercise), at work (e.g., walking or lifting as part of work requirements), for travel (e.g., walking or riding a bike for transport) and domestically (e.g., household chores). Evidence suggests that the domains in which physical activity occurs affect the relationship between physical activity and depression [12]. McKercher et al. found that 1.25 hours per week of leisure-time physical activity resulted in a 45% reduction in depression whilst more than 10 hours a week of work-related physical activity resulted in twofold higher levels of depression compared to a sedentary group [12]. Additionally, White et al. found that transport-related physical activity and leisure-time physical activity resulted in lower levels of depression [13], whilst work-related physical activity was associated with higher levels of depression, echoing the findings by McKercher et al. [12] Furthermore, in a study with 14,381 adults, Schuch et al. found that even small amounts of leisure-time physical activity were beneficial [14].

Although the association between physical activity and depression has been well established, further research is required to understand what factors may moderate this relationship between physical activity and depression [15]. Previous studies suggest that potential moderators for the association between depression and physical activity may include demographical variables (e.g., age, sex, marital status), psychological traits (e.g., personality traits or characteristics) and social and environmental factors (e.g., socioeconomic status, ethnicity) [16–20]. Studies also suggested that the relationship between physical activity and depression could differ for people in different Body Mass Index (BMI) categories. Cho et al. found that people who were underweight and inactive had an increased risk of depression compared to active, healthy weight individuals [21]. Vallance et al. found that higher levels of MVPA resulted in less depressive symptoms for or overweight/obese people but not for healthy weight adults, showing that BMI moderated the association between physical activity and depression [22]. On the other hand, Schuch et al. examined the effect of exercise added to the treatment of severely depressed participants and found that BMI did not moderate the effects of physical activity on depression [23]. Given inconsistent evidence and the small number of studies in this area, further research is required. Additionally, the moderation effect of BMI on the relationship between domain-specific physical activity and depression is currently unknown.

Therefore, the purpose of this study was to investigate: 1) the relationship between total and domain-specific (leisure, work, and travel) physical activity and depression; 2) the relationship between BMI categories (underweight/healthy, overweight, and obese) and depression; 3) the moderating effect of BMI categories on the relationship between total and domain-specific physical activity and depression. Findings from this study will improve the understanding of the associations of domain-specific physical activity, BMI, and depression and as a result, inform resource allocations for interventions targeting people in different weight groups. In addition, the findings assist with setting priorities and selection of suitable intervention strategies for specific domains of physical activity to maximize the effectiveness of mental health interventions.

## Methods

### Participants and recruitment

Data from the 2011-2012 and 2013-2014 National Health and Nutrition Examination Survey (NHANES) were used. NHANES is a cross-sectional survey that uses complex, multistage probability sampling to recruit a non-institutionalised, nationally representative sample of the US population. NHANES selects participants from approximately 15 counties (5000 participants) across the U. S per year [24]. The National Centre for Health Statistics Ethics Review Board approved the NHANES survey protocols (Protocol #2011-2017) [25], and the study complied with the Declaration of Helsinki. All participants gave written informed consent.

Once selected for the NHANES study, participants were sent an introductory letter and received an in-person visit for a household screener to confirm eligibility. To obtain a wide range of health data, an interviewer conducted a survey interview in-home, and the participants attended a mobile health examination centre for physical measurements, dental examination, and collection of specimens for laboratory testing. The 2011-2012 NHANES cycle interviewed a total of 9,756 people with a 72.6% response rate and examined 9,338 people with a 69.5% response rate [26]. The 2013-2014 survey interviewed 10,175 people with a 71% response rate and examined 9,813 people with a 68.5% response rate [27]. Combined, the 2011-2012 and 2013-2014 NHANES cycles included 19,931 participants.

The current study included all adults aged 20 years and older. As such, 8,602 participants under 20 years were excluded. Pregnant women (*n* = 122) and participants who required special equipment to walk (*n* = 1,161) were also excluded from the study due to their limited capacity for physical activity and how these aspects may affect BMI and depression. The final subsample included 10,047 participants.

### Measures

#### Depression

The Patient Health Questionnaire (PHQ-9) [28] was used to measure depression. The PHQ-9 has a high validity and reliability with Cronbach alphas of .86 and .89, indicating sound psychometric properties [29–31]. The measure was completed during the health examination at the mobile centre by trained interviewers using the Computer-Assisted Personal Interviewing (CAPI) system. Participants were asked about the frequency of symptoms of depression in the last 2 weeks using a nine-item screening instrument. The items in PHQ-9 reflect the criteria to assess major depressive disorder and include questions relating to mood, interest/pleasure, sleep problems, fatigue, appetite changes, guilt, difficultly concentrating, psychomotor changes and suicidal tendencies, as per the diagnosis criteria in DSM-5 [32]. An example of PHQ-9 questions includes “Over the last 2 weeks, how often have you been bothered by the following problems: 1) little interest or pleasure in doing things? 2) feeling down, depressed, or hopeless? 3) trouble falling or staying asleep, or sleeping too much? Available responses included "not at all," "several days," "more than half the days," and "nearly every day", and responses were given a point ranging from 0 to 3. No questions were reverse scored. Scores were summed to give a total score ranging from 0 to 27, with higher scores indicating higher levels of depression. Mild, moderate, and severe depressive symptoms are indicated by scores of 5, 10 and 20, respectively. Scores higher than 10 indicate major depressive disorder [33].

#### Body-mass index

BMI was calculated as weight in kilograms divided by height in meters squared. NHANES participants attended the mobile examination center and had their body measurements collected by a trained health technician. Standardized protocol and calibrated instruments were used in data collection [34]. BMI were categorised as underweight (BMI below 18.5), healthy weight (BMI 18.5 – 24.9), overweight (BMI 25 – 29.9), obese (BMI above 30.0). Due to the underweight category having a low number of participants (*n* = 168), underweight and healthy weight participants were grouped together in the analysis.

#### Physical activity

Participants completed the Global Physical Activity Questionnaire (GPAQ) [35] at home using the Computer-Assisted Personal Interview system. The GPAQ was developed by WHO to collect information regarding sedentary information as well as the minutes spent per week engaging in physical activity in three domains (leisure, work, and travel) and at different intensities (moderate such as brisk walking or cycling and vigorous such as running or football). The self-report measure contained 16 questions. For the current study, total MVPA and MVPA in each domain (vigorous physical activity minutes were doubles) was further categorised into dichotomous variables with a cut-point of 150 MVPA min/week, as recommended by the physical activity guidelines [11]. The validity and reliability of GPAQ have been tested across multiple studies (including NHANES) and found to be an acceptable instrument for monitoring physical activity [36–39].

#### Covariates

Age, gender, ethnicity, marital status, education and poverty ratio were controlled for in all models of the analyses as studies have found that these variables influence the relationship between physical activity and depression and are associated with both variables [14, 40–44]. Gender was either “male” or “female”. Ethnicity was grouped into “Non-Hispanic White”, “Non-Hispanic Black”, “Non-Hispanic Asian”, and “Mexican American/others”, with others including multi-ethnic. The categories for marital status included “never married”, “married/ with partner”, or “other”, which included participants that were widowed, divorced, or separated. Education was categorised into “high school or below,” which included less than 9^th^ grade, 9-11^th^ grade (includes 12^th^ grade with no diploma and high school graduate/GED or equivalent) and “above high school”, which included some college or associate degree and college graduate or above. Poverty income ratio was a continuous variable and is a ratio of family income-to-poverty threshold, dependent on household size. This variable measures household income in comparison to the poverty line to determine the level of poverty for the household. A ratio of 1 means that the household income and poverty level are the same, whereas if the ratio is less than 1, it means that household income is less than the poverty line level. The poverty ratio range for the current study was 0-5.

### Statistical analysis

Data analysis was undertaken using SPSS, Complex Samples module (version 28; SPSS IBM Company) and SPSS survey procedures were used to account for complex survey design. Taylor series linearisation was chosen as a variance estimation method as recommended by NHANES [45]. As two NHANES cycles were combined, two-year weights were divided by two to recalculate four-year weights as instructed by NHANES. Four-year examination weights were used for analyses.

As depression data were missing for more than 10% of the population, a regression method was used to impute missing values based on age, gender, ethnicity, marital status, education, and poverty income ratio. The standard deviation of the original depression variable was matched to that of the imputed variable by adding random normal variability. Missing values for other variables ranged from 0.1% to 8.2%. Analyses were run using original and imputed data. As results were consistent only those from original data were presented. Data were checked to ensure there were no outliers, and that data matched NHANES data documents.

Weighted percentages and means with standard errors (SE) were generated as descriptive statistics. Complex sample linear regression was used to analyse the relationship between depression and meeting or not meeting 150 minutes/week of total MVPA, workplace MVPA, leisure MVPA, and travel MVPA. This cut-off point was for the total MVPA making it more difficult to meet the recommendations for a single component; therefore, weighted averages of depression scores for each component were also presented as a sensitivity check (Supplemental Table 1). Further analyses examined the relationship between depression as the outcome variable and BMI with three categories (underweight/healthy, overweight, and obese (reference)) as the independent variable. The moderation effect of BMI on the association between physical activity and depression was also analysed using complex sample linear regression. Both bivariate and multivariable analyses adjusted for age, gender, ethnicity, education level, marital status, and poverty income ratio were conducted. Due to multiple comparisons between weight groups, Bonferroni post-hoc adjustment was applied. Two-sided p-values were used and considered significant if <0.05.

## Results

Sample characteristics are presented in Table 1. Participants’ average age was 46.4 years (SE = .47), with 50.8% of participants being female. A total of 641 participants, equaling 7.01%, met the criteria for having depression (score of 10 or greater) and the average depression score was 2.80 (SE = 0.08). Approximately one third of participants were underweight/healthy weight (30.8%), overweight (34.0%) or obese (35.2%). About two thirds (65.3%) of participants engaged in 150 or more minutes of total physical activity whilst 33.2%, 39.4% and 15.6% of participants engaged in at least 150 minutes of work, leisure and transport physical activity, respectively. The sample characteristics between the two NHANES cycles were similar. Weighted averages and percentages of demographic characteristics for participants with depression scores <10 and ≥10 were presented in Supplemental Table 2.

**Table 1:** Weighted sample characteristics

	Cycle 2011-2012		Cycle 2013-2014		Total	
	N	M or % (SE)	N	M or % (SE)	N	M or % (SE)
Age (years)	4935	46.48 (0.85)	5112	46.40 (0.40)	10047	46.44 (0.47)
Gender						
Male	2493	49.3% (0.9)	2491	49.1% (0.5)	4984	49.2% (0.5)
Female	2442	50.7% (0.9)	2621	50.9% (0.5)	5063	50.8% (0.5)
Marital Status						
Never married	1102	20.3% (2.5)	1019	19.5% (1.3)	2121	19.9% (1.4)
Married/with partner	2856	62.4% (2.1)	3078	63.0% (1.3)	5934	62.7% (1.3)
Others	973	17.3% (0.8)	1014	17.5% (0.6)	1987	17.4% (0.5)
Education						
High school or below	2134	35.6% (2.6)	2181	36.1% (2.2)	4315	35.9% (1.7)
Above high school	2799	64.4% (2.6)	2927	63.9% (2.2)	5726	64.1% (1.7)
Ethnicity						
Non-Hispanic White	1800	66.9% (3.8)	2171	65.8% (3.3)	3971	66.4% (2.5)
Non-Hispanic Black	1248	11.0% (2.1)	999	10.9% (1.6)	2247	10.9% (1.3)
Non-Hispanic Asian	747	5.3% (0.9)	634	5.6% (0.7)	1381	5.5% (0.6)
Mexican American/others	1140	16.7% (2.4)	1308	17.8% (2.5)	2448	17.3% (1.7)
Poverty income ratio	4518	2.95 (0.11)	4704	2.98 (0.11)	9222	2.97 (0.08)
Total MVPA						
<150min/week	1783	33.1% (1.7)	1932	36.4% (0.9)	3715	34.7% (0.9)
≥150min/week	3150	66.9% (1.7)	3180	63.6% (0.9)	6330	65.3% (0.9)
Work MVPA						
<150min/week	3460	67.4% (1.3)	3470	66.2% (1.2)	6930	66.8% (0.9)
≥150min/week	1464	32.6% (1.3)	1631	33.8% (1.2)	3095	33.2% (0.9)
Leisure MVPA						
<150min/week	3114	59.3% (2.2)	3275	61.9% (1.1)	6389	60.6% (1.2)
≥150min/week	1816	40.7% (2.2)	1835	38.1% (1.1)	3651	39.4% (1.2)
Transport MVPA						
<150min/week	3964	81.3% (1.7)	4370	87.5% (1.0)	8334	84.4% (1.0)
≥150min/week	963	18.7% (1.7)	736	12.5% (1.0)	1699	15.6% (1.0)
BMI						
Underweight/healthy	1541	31.5% (1.8)	1534	30.2% (0.9)	3075	30.8% (1.0)
Overweight	1555	34.5% (1.5)	1615	33.4% (0.8)	3170	34.0% (0.9)
Obese	1603	34.0% (1.3)	1778	36.4 (0.9)	3381	35.2% (0.8)
Depression scores	4143	2.74 (0.13)	4504	2.85 (0.09)	8647	2.80 (0.08)

M Mean score, *SE* Standard error, *MVPA* Moderate-vigorous physical activity

Table 2 shows the association between total and domain-specific PA and BMI with depression. Participants who met 150 min/week recommended guidelines for total physical activity, on average, had fewer depression symptoms than participants who did not meet the guidelines (B = 0.95, 95% CI [0.66, 1.25], adjusted B = 0.83, 95% CI [0.50, 1.16], *p*-values<0.001). Participates who completed equal to or greater than 150 min/week of leisure physical activity had significantly fewer depression symptoms than participants who completed less than 150 min/week (B = 1.04, 95% CI [0.79, 1.29], adjusted B = 0.84, 95% CI [0.57, 1.11], *p*-values<0.001), indicating that leisure MVPA was a predictor for the depression score. Work-related and travel related MVPA were not significant predicters of depression in either the bivariate or multivariable models. In addition, overweight (B = -0.62, 95% CI [-0.97, -0.26], *p*-value<0.001, adjusted B = -0.40, 95% CI [-0.76, -0.04], *p*-value = 0.026) and underweight/normal weight participants (B = -0.59, 95% CI [-0.95, -0.22], adjusted B = -0.60, 95%CI [-0.96, -0.25], *p*-values<0.001) experienced less depressive symptoms compared to obese participants.

**Table 2:** Associations between physical activity domains and BMI with depression

	Model 1 B [95% CI]	Model 2 B [95% CI]
Total MVPA (<150 vs ≥150 minutes)	0.95 [0.66, 1.25]^***^	0.83 [0.50, 1.16]^***^
Leisure MVPA (<150 vs ≥150 minutes)	1.04 [0.79, 1.29]^***^	0.84 [0.57, 1.11]^***^
Work-related MVPA (<150 vs ≥150 minutes)	0.13 [-0.12, 0.38]	0.16 [-0.10, 0.42]
Travel MVPA (<150 vs ≥150 minutes)	0.14 [-0.26, 0.55]	0.26 [-0.16, 0.67]
BMI (underweight/normal vs obese)	-0.59 [-0.95, -0.22]^***^	-0.60 [-0.96, -0.25]^***^
BMI (overweight vs obese)	-0.62 [-0.97, -0.26]^***^	-0.40 [-0.76, -0.04]^*^

Model 1: bivariate model, include only physical activity and depression Model 2: multivariable model, adjusted for age, gender, ethnicity, education, marital status, and poverty-income-ratio.

^*^p<0.05, ^**^p<0.01, ^***^p,0.001

The moderation effect of BMI on the association between total MVPA and depression was not statistically significant in both bivariate (*p* = 0.814) and multivariable analyses (*p* = 0.991) meaning that the relationship between total MVPA and depression did not differ based on BMI category. Likewise, no moderation effects of BMI on the association between physical activity and depression were found for leisure-time, work-related and travel related physical activity.

## Discussion

The findings show that depression and physical activity were found to have a negative association, as participants who engaged in at least 150 minutes of total physical activity per week experienced fewer depressive symptoms than people who did not, which is consistent with previous research [8]. It was found that the domain in which physical activity occurs directly influences the beneficial aspects of physical activity on depression. Participants who met 150 minutes per week of leisure-time physical activity experienced fewer depressive symptoms than those that did not meet 150 minutes per week, which is similar to previous findings [12, 14]. However, there was no significant relationship between physical activity and depression for work- or travel-related physical activity, which is inconsistent with findings by White et al. [13], who previously demonstrated that travel-related physical activity was associated with depression. The results indicate that leisure-time physical activity may have more benefits in reducing depression than work- or travel-related physical activity.

There are several possible mechanisms for this domain effect of physical activity on depression. Firstly, leisure-time physical activity is more likely to be viewed as a self-directed, enjoyable task compared to physical activity in other domains [13]. According to the self-determination theory, autonomous motivation associated with physical activity is more likely to produce psychological satisfaction (autonomy, competence, and relatedness), which in turn supports mental health [46]. Participation in physical activity due to enjoyment is associated with autonomous and intrinsic motivation [47]. Thus, leisure-time physical activity is more likely to be undertaken for enjoyment rather than work- or travel-related physical activity, as these are often compulsory tasks or done for external rewards which are associated with extrinsic rather than intrinsic motivation and thus have less benefits for mental health [47].

Secondly, the distraction hypothesis suggests that physical activity offers a distraction from stressful life events, which supports the reduction of depression [48]. Leisure-time physical activity has potential distraction benefits, whereas travel- and work-related physical activity, which is performed in places that are potentially stressful, do not hold the same distraction benefits. Asztalos et al. also found that physical activity related to travelling to work can be associated with increased stress amongst blue-collar workers, who may choose active travelling due to economy factor rather than enjoyment [49]. Thus, travel-related physical activity could be unenjoyable for some.

Thirdly, travel- and potentially work-related physical activity are most likely to be done alone, making it less likely to improve a person’s sense of belonging, whereas leisure-time physical activity often incorporates social interaction, which benefits mental health [50]. Physical activity is also noted to elicit feelings of self-mastery and self-efficacy by completing tasks that an individual finds challenging [51, 52]. As work- and travel-related physical activity are more likely to only involve activities such as walking, these domains may hold fewer opportunities for self-mastery for physical activity [53], which is associated with psychological wellbeing.

Lastly, the increase of neurotransmitters such as endorphins is also a plausible mechanism for the physical activity and depression relationship [54]. Enjoyable physical activity has been noted to increase the production of the neurotransmitter serotonin, which regulates mood and stress [55]. Thus, if travel- and work-related physical activity is not seen as enjoyable, it may not have the same serotonin production as leisure-time physical activity, therefore not allowing for the same levels of serotonin and reduction in depressive symptoms.

In line with previous research, BMI was found to have a positive association with depression, in that as BMI increased, so too did depression levels [56, 57]. This may be due to low body weight being idealized in the US, resulting in individuals in the overweight or obese BMI categories potentially experiencing increased body dissatisfaction and self-esteem issues, which are noted to increase risks of depression [58]. Previous studies have also noted that the perception of being overweight has an effect on mental ill health [59]. Further, the relationship between depression and BMI has been found to be bi-directional [60]. Depression may lead to changes in lifestyle such as lack of exercise, insufficient sleep, increase in fatigue levels and unhealthy eating habits, which could lead to weight gain [61]. Depression can also result in disturbances in cortisol levels and dysregulation of the hypothalamic-pituitary-adrenal axis, which is noted to result in weight gain [62, 63]. Antidepressant medication has also been shown to induce weight gain [64].

Finally, it was found that BMI did not affect the relationship between domain-specific physical activity and depression. Participants who completed 150 minutes per week of physical activity, regardless of BMI category, experienced reduced depression scores compared to those that did not complete 150 minutes per week. Thus, physical activity is therefore beneficial for individuals regardless of BMI category in reducing depression, which means that the promotion of physical activity should cater to all BMI categories for both mental and physical health benefits. The moderating effect of BMI in previous research has been inconsistent. One study using accelerometers found a moderation effect of BMI in the U.S. population, i.e., there was a significant association between MVPA and depression in overweight/obese adults but no association in healthy weight adults [22]; however, a systematic review among people with major depressive disorder did not find moderation effects for any demographic factors including BMI [65]. Although BMI in the current study was not a moderator for the association between physical activity and depression, the esteem-boosting properties of physical activity [66] could be beneficial for people in higher BMI categories who may experience low self-esteem [58]. As low levels of self-esteem were associated with depression [67], increase in self-esteem may result in improvement in depression among people with higher BMI categories. Other studies have also shown that physical activity has many health benefits for overweight people, highlighting the benefit of being fit and overweight [68]. Ortega et al. noted that obese and metabolically healthy individuals tend to have higher physical activity rates and fewer concerns for cardiovascular disease and mortality compared to obese and metabolically unhealthy individuals [68].

The strengths of the current study included a large population-based representative sample which allows for findings to be generalized. NHANES also has a high response rate of 71.5% average for the two cycles used within the study. Further, validated depression and physical activity tools were used within the survey and BMI was objectively measured. However, the study design was cross-sectional, which limited the study ability to infer causality [69]. Despite using validated self-report measures, there is a potential for recall bias. Self-report physical activity questionnaires may over-estimate the amount of physical activity although they are deemed to be one of the most suitable options to measure domain-specific physical activity [14]. Advancements in technology may present suitable options such as combining GPS and accelerometer data for future studies [70, 71].

In conclusion, the relationship between physical activity and depression was found to be domain-specific, in that leisure-time physical activity was associated with lower depressive symptoms. Further, higher BMI was associated with depression, but BMI did not moderate the relationship between domain-specific physical activity and depression. As such, interventions that encourage individuals to specifically engage in leisure-time physical activity may be best suited to improve mental health and depression, as compared to interventions focusing on overall, work-related or travel-related physical activity. Having activities to improve participant’s weight status may also help increase the effectiveness of these interventions. However, studies that were purposefully designed to examine causal relationships between different types of physical activity and depression are needed to confirm the findings in this study. Investigating the roles of residential and work environments, type of work, and psychosocial factors in the associations between physical activity, weight status, and depression may be necessary to provide a better understanding of these relationships. Future studies may also examine ways to improve the benefits of work and travel physical activity for depression through increased enjoyment or social interaction within the activity.

## Supplementary Information

**Additional file 1 : Table S1.** Weighted averages of depression scores for each MVPA groups. **Table S2.** Weighted averages and percentages of characteristics by depression.

## Data Availability

This study used public data at https://www.cdc.gov/nchs/nhanes/index.htm

## References

[1] World Health Organisation. Depression. 2020 [cited 2021 30th April]; Available from: https://www.who.int/news-room/fact-sheets/detail/depression.

[2] Moreno-Agostino D, Wu Y-T, Daskalopoulou C, Hasan MT, Huisman M, Prina M (2021). Global trends in the prevalence and incidence of depression:a systematic review and meta-analysis. J Affect Disord..

[3] Iranpour S, Sabour S, Koohi F, Saadati HM (2022). The trend and pattern of depression prevalence in the U.S.: Data from National Health and Nutrition Examination Survey (NHANES) 2005 to 2016. J Affect Disord..

[4] Weinberger AH, Gbedemah M, Martinez AM, Nash D, Galea S, Goodwin RD (2017). Trends in depression prevalence in the USA from 2005 to 2015: widening disparities in vulnerable groups. Psychol Med..

[5] Malhi GS, Bassett D, Boyce P, Bryant R, Fitzgerald PB, Fritz K, et al. Royal Australian and New Zealand College of Psychiatrists clinical practice guidelines for mood disorders. Aust N Z J Psychiatry. 2015;49(12):1087-206. 10.1177/0004867415617657 26643054

[6] Cooney GM, Dwan K, Greig CA, Lawlor DA, Rimer J, Waugh FR, McMurdo M, Mead GE. Exercise for depression. Cochrane Database Syst Rev. 2013;(9). 10.1002/14651858.CD004366.pub6 PMC9721454 24026850

[7] Rethorst CD, Wipfli BM, Landers DM (2009). The antidepressive effects of exercise: A meta-analysis of randomized trials. Sports Med..

[8] Rebar A, Stanton R, Geard D, Short C, Duncan M, Vandelanotte C (2015). A meta-meta-analysis of the effect of physical activity on depression and anxiety in non-clinical adult populations. Health Psychol Rev..

[9] Bailey A, Hetrick S, Rosenbaum S, Purcell R, Parker A (2018). Treating depression with physical activity in adolescents and young adults: A systematic review and meta-analysis of randomised controlled trials. Psychol Med..

[10] Mason P, Curl A, Kearns A (2016). Domains and levels of physical activity are linked to adult mental health and wellbeing in deprived neighbourhoods: A cross-sectional study. Mental Health Phys Act.

[11] U.S. Department of Health and Human Services (2018). Physical Activity Guidelines for Americans.

[12] McKercher CM, Schmidt MD, Sanderson KA, Patton GC, Dwyer T, Venn AJ. Physical activity and depression in young adults. Am J Prev Med. 2009;36(2):161-4. 10.1016/j.amepre.2008.09.036 19062235

[13] White R, Babic M, Parker P, Lubans D, Astell-Burt T, Lonsdale C (2017). Domain-specific physical activity and mental health: a meta-analysis. Am J Prev Med..

[14] Schuch FB, AeO W, Vancampfort D, Stubbs B, Teychene M, Lotufo PA (2021). Cross-sectional associations of leisure and transport related physical activity with depression and anxiety. J Psychiatr Res..

[15] Pickett K, Kendrick T, Yardley L (2017). “A forward movement into life”: A qualitative study of how, why and when physical activity may benefit depression. Mental Health Phys Act.

[16] Button KS, Turner N, Campbell J, Kessler D, Kuyken W, Lewis G (2015). Moderators of response to cognitive behavioural therapy as an adjunct to pharmacotherapy for treatment-resistant depression in primary care. J Affect Disord..

[17] Donker T, Batterham PJ, Warmerdam L, Bennett K, Bennett A, Cuijpers P (2013). Predictors and moderators of response to internet-delivered Interpersonal Psychotherapy and Cognitive Behavior Therapy for depression. J Affect Disord..

[18] MacPherson HA, Algorta GP, Mendenhall AN, Fields BW, Fristad MA (2014). Predictors and moderators in the randomized trial of multifamily psychoeducational psychotherapy for childhood mood disorders. J Clin Child Adolesc Psychol..

[19] Ozomaro U, Wahlestedt C, Nemeroff CB (2013). Personalized medicine in psychiatry: problems and promises. BMC Med..

[20] Papakostas GI, Fava M (2008). Predictors, moderators, and mediators (correlates) of treatment outcome in major depressive disorder. Dialogues Clin Neurosci..

[21] Cho J, Jin Y, Kang H (2018). Weight status, physical activity, and depression in Korean older adults. J Epidemiol..

[22] Vallance JK, Winkler EAH, Gardiner PA, Healy GN, Lynch BM, Owen N (2011). Associations of objectively-assessed physical activity and sedentary time with depression: NHANES (2005–2006). Prev Med..

[23] Schuch FB, Vasconcelos-Moreno MP, Borowsky C, Zimmermann AB, Rocha NS, Fleck MP (2015). Exercise and severe major depression: Effect on symptom severity and quality of life at discharge in an inpatient cohort. J Psychiatr Res..

[24] Chen T-C, Parker JD, Clark J, Shin H-C, Rammon JR, Burt VL (2018). National health and nutrition examination survey: estimation procedures.

[25] National Center for Health Statistics (2017). NCHS Research Ethics Review Board (ERB) Approval.

[26] Centers for Disease Control and Prevention (CDC), National Center for Health Statistics (NCHS) (2012). Unweighted Response Rates for NHANES 2011-2012 by Age and Gender.

[27] Centers for Disease Control and Prevention (CDC), National Center for Health Statistics (NCHS) (2014). Unweighted Response Rates for NHANES 2013-2014 by Age and Gender.

[28] Kroenke K, Spitzer R (2002). The PHQ-9: A new depression diagnostic and severity measure. Psychiatric Annals..

[29] Kocalevent R-D, Hinz A, Brähler E (2013). Standardization of the depression screener patient health questionnaire (PHQ-9) in the general population. Gen Hosp Psychiatry..

[30] Kroenke K, Spitzer RL, Williams JB (2001). The PHQ-9: validity of a brief depression severity measure. J Gen Intern Med..

[31] Monahan PO, Shacham E, Reece M, Kroenke K, Ong’or WO, Omollo O (2008). Validity/reliability of PHQ-9 and PHQ-2 depression scales among adults living with HIV/AIDS in western Kenya. J Gen Intern Med..

[32] American Psychiatric Association (2013). Diagnostic and statistical manual of mental disorders : DSM-5.

[33] Kroenke K, Spitzer R, Williams J, Lowe B (2010). The patient health questionnaire somatic, anxiety, and depressive symptom scales: A systematic review. Gen Hosp Psychiatry..

[34] National Center for Health Statistics. National health and nutrition examination survey anthropometry procedures manual: Centers for Disease Control and Prevention; 2016.

[35] World Health Organization. Global Physical Activity Questionnaire (GPAQ) Analysis Guide. Surveillance and Population-Based Prevention. Prevention of Noncommunicable Diseases Department. Geneva: World Health Organization.

[36] Bull F, Maslin T, Armstrong T (2009). Global Physical Activity Questionnaire (GPAQ): Nine country reliability and validity study. J Phys Act Health.

[37] Cleland CL, Hunter RF, Kee F, Cupples ME, Sallis JF, Tully MA (2014). Validity of the global physical activity questionnaire (GPAQ) in assessing levels and change in moderate-vigorous physical activity and sedentary behaviour. BMC Public Health..

[38] Herrman SD, Heumann KJ, Ananian CAD, Ainsworth BE (2013). Validity and reliability of the global physical activity questionnaire (GPAQ). Measure Phys Educ Exerc Sci.

[39] Gore S, Goldberg A, Huang MH, Shoemaker M, Blackwood J (2020). Validity of the global physical activity questionnaire in older adults with chronic obstructive pulmonary disease: Results from the national health and nutrition examination survey. Cardiopulm Phys Ther J..

[40] King A, Blair S, Bild D, Dishman R, Dubbert P, Marcus B (1992). Determinants of physical activity and interventions in adults. Med Sci Sports Exerc..

[41] Parks S, Housemann R, Brownson R (2003). Differential correlates of physical activity in urban and rural adults of various socioeconomic backgrounds in the United States. J Epidemiol Commun Health..

[42] Ranjbar E, Memari AH, Hafizi S, Shayestehfar M, Mirfazeli FS, Eshghi MA (2015). Depression and exercise: A clinical review and management guideline. Asian. J Sports Med..

[43] Troiano R, Berrigan D, Dodd K, Mâsse L, Tilert T, McDowell M (2008). Physical activity in the United States measured by accelerometer. Med Sci Sports Exerc..

[44] Zhang J, Yen ST (2015). Physical activity, gender difference, and depressive symptoms. Health Serv Res..

[45] Centers for Disease Control and Prevention (CDC), National Center for Health Statistics (NCHS) (2018). National Health and Nutrition Examination Survey: Analytic Guidelines, 2011-2014 and 2015-2016.

[46] Weinstein N, Ryan RM (2010). When helping helps: Autonomous motivation for prosocial behavior and its influence on well-being for the helper and recipient. J Pers Soc Psychol..

[47] Ryan RM, Deci EL (2000). Intrinsic and extrinsic motivations: classic definitions and new directions. Contemp Educ Psychol..

[48] Leith LM (2010). Foundations of exercise and mental health.

[49] Asztalos M, Wijndaele K, De Bourdeaudhuij I, Philippaerts R, Matton L, Duvigneaud N (2009). Specific associations between types of physical activity and components of mental health. J Sci Med Sport..

[50] Bailey M, McLaren S (2005). Physical activity alone and with others as predictors of sense of belonging and mental health in retirees. Aging Ment Health..

[51] Craft LL, Perna FM (2004). The benefits of exercise for the clinically depressed. Prim Care Companion J Clin Psychiatr..

[52] Paluska SA, Schwenk TL (2000). Physical activity and mental health. Sports Med..

[53] Sohn AJ, Hasnain M, Sinacore JM (2007). Impact of exercise (walking) on blood pressure levels in African American adults with newly diagnosed hypertension. Ethn Dis..

[54] Phillips C, Fahimi A. Immune and Neuroprotective Effects of Physical Activity on the Brain in Depression. Front Neurosci. 2018;12:498. 10.3389/fnins.2018.00498 PMC6070639 30093853

[55] Ernst C, Olson AK, Pinel JPJ, Lam RW, Christie BR (2006). Antidepressant effects of exercise: evidence for an adult-neurogenesis hypothesis?. J Psychiatry Neurosci..

[56] Bjerkeset O, Romundstad P, Evans J, Gunnell D (2007). Association of adult body mass index and height with anxiety, depression, and suicide in the general population. Am J Epidemiol..

[57] De Wit LM, Van Straten A, Van Herten M, Penninx BW, Cuijpers P. Depression and body mass index, a u-shaped association. BMC Public Health. 2009;9(1):1-6. 10.1186/1471-2458-9-14 PMC2631467 19144098

[58] Hoek HW, Harten PNv, Hermans KME, Katzman MA, Matroos GE, Susser ES. (2005). The Incidence of Anorexia Nervosa on Curaçao. Am J Psychiatry..

[59] Atlantis E, Ball K (2008). Association between weight perception and psychological distress. Int J Obes..

[60] Konttinen H, Kiviruusu O, Huurre T, Haukkala A, Aro H, Marttunen M (2014). Longitudinal associations between depressive symptoms and body mass index in a 20-year follow-up. Int J Obes (Lond)..

[61] Luppino FS, de Wit LM, Bouvy PF, Stijnen T, Cuijpers P, Penninx BWJH (2010). Overweight, obesity, and depression: A systematic review and meta-analysis of longitudinal studies. Arch Gen Psychiatry..

[62] Björntorp P (1996). The regulation of adipose tissue distribution in humans. Int J Obes Relat Metab Disord.

[63] Bornstein SR, Schuppenies A, Wong ML, Licinio J (2006). Approaching the shared biology of obesity and depression: the stress axis as the locus of gene-environment interactions. Mol Psychiatry..

[64] Stunkard AJ, Faith MS, Allison KC (2003). Depression and obesity. Biol Psychiatry..

[65] Schuch F, Dunn A, Kanitz A, Delevatti R, Fleck M (2016). Moderators of response in exercise treatment for depression: A systematic review. J Affect Disord..

[66] Stewart AL, Hays RD, Wells KB, Rogers WH, Spritzer KL, Greenfield S (1994). Long-term functioning and well-being outcomes associated with physical activity and exercise in patients with chronic conditions in the medical outcomes study. J Clin Epidemiol..

[67] Sowislo JF, Orth U (2013). Does low self-esteem predict depression and anxiety? A meta-analysis of longitudinal studies. Psychol Bull.

[68] Ortega FB, Lee D-c, Katzmarzyk PT, Ruiz JR, Sui X, Church TS (2012). The intriguing metabolically healthy but obese phenotype: cardiovascular prognosis and role of fitness. Eur Heart J..

[69] Kirk RE (2013). Experimental design: Procedures for the behavioral sciences.

[70] Duncan MJ, Badland HM, Mummery WK (2009). Applying GPS to enhance understanding of transport-related physical activity. J Sci Med Sport..

[71] Rodriguez DA, Cho G-H, Elder JP, Conway TL, Evenson KR, Ghosh-Dastidar B (2012). Identifying walking trips From GPS and accelerometer data in adolescent females. J Phys Act Health..

