# Supplementary material for: Associations between depression, domain-specific physical activity, and BMI among US adults: NHANES 2011-2014 cross-sectional data
Source: BMC Public Health. 2022 Aug 25;22:1618. doi: 10.1186/s12889-022-14037-4 (PMC9413906; doi:10.1186/s12889-022-14037-4)
Supplement: Supplementary file 1 — Additional file 1 : Table S1. Weighted averages of depression scores for each MVPA groups. Table S2. Weighted averages and percentages of characteristics by depression. [file 12889_2022_14037_MOESM1_ESM.docx]

| Table S1. Weighted averages of depression scores for each MVPA groups | | |
| --- | --- | --- |
|  | n | Mean (SE) |
| Leisure MVPA |  |  |
| <50 min/week | 4287 | 3.38 (0.11) |
| 50 - <100 min/week | 618 | 2.72 (0.16) |
| 100 - <150 min/week | 542 | 2.61 (0.19) |
| ≥150 min/week | 3195 | 2.17 (0.09) |
| Work MVPA |  |  |
| <50 min/week | 5401 | 2.84 (0.10) |
| 50 - <100 min/week | 256 | 2.85 (0.38) |
| 100 - <150 min/week | 221 | 2.75 (0.31) |
| ≥150 min/week | 2751 | 2.71 (0.10) |
| Transport MVPA |  |  |
| <50 min/week | 6383 | 2.86 (0.08) |
| 50 - <100 min/week | 460 | 2.64 (0.20) |
| 100 - <150 min/week | 350 | 2.27 (0.22) |
| ≥150 min/week | 1442 | 2.67 (0.19) |

| Table S2. Weighted averages and percentages of characteristics by depression. | | | | |
| --- | --- | --- | --- | --- |
|  | Depression scores <10 | | Depression scores ≥10 | |
|  | N | M or % (SE) | N | M or % (SE) |
| Age (years) | 8006 | 46.84 (0.52) | 641 | 45.19 (0.81) |
| Gender |  |  |  |  |
| Male | 4130 | 51.08 (0.59) | 222 | 35.19 (2.45) |
| Female | 3876 | 48.92 (0.59) | 419 | 64.81 (2.45) |
| Marital Status |  |  |  |  |
| Never married | 1677 | 19.51 (1.50) | 152 | 25.06 (1.68) |
| Married/with partner | 4812 | 63.88 (1.31) | 279 | 46.69 (2.20) |
| Others | 1514 | 16.61 (0.53) | 210 | 28.25 (2.01) |
| Education |  |  |  |  |
| High school or below | 3249 | 33.96 (1.89) | 358 | 50.15 (2.55) |
| Above high school | 4755 | 66.04 (1.89) | 283 | 49.85 (2.55) |
| Ethnicity |  |  |  |  |
| Non-Hispanic White | 3272 | 67.93 (2.47) | 285 | 66.84 (3.45) |
| Non-Hispanic Black | 1785 | 10.4 (1.24) | 133 | 10.96 (1.80) |
| Non-Hispanic Asian | 1039 | 5.10 (0.54) | 27 | 1.76 (0.32) |
| Mexican American/others | 1910 | 16.57 (1.75) | 196 | 20.43 (2.41) |
| Poverty income ratio | 7428 | 3.06 (0.07) | 589 | 2.09 (0.14) |
| Total MVPA |  |  |  |  |
| <150min/week | 2812 | 32.74 (0.92) | 320 | 50.04 (2.52) |
| ≥150min/week | 5194 | 67.26 (0.92) | 321 | 49.96 (2.52) |
| Work MVPA |  |  |  |  |
| <150min/week | 5432 | 65.43 (0.99) | 446 | 70.11 (2.15) |
| ≥150min/week | 2560 | 34.57 (0.99) | 191 | 29.89 (2.15) |
| Leisure MVPA |  |  |  |  |
| <150min/week | 4945 | 58.72 (1.37) | 502 | 78.06 (2.03) |
| ≥150min/week | 3056 | 41.28 (1.37) | 139 | 21.94 (2.03) |
| Transport MVPA |  |  |  |  |
| <150min/week | 6647 | 84.53 (0.97) | 546 | 85.87 (2.22) |
| ≥150min/week | 1349 | 15.47 (0.97) | 93 | 14.13 (2.22) |
| BMI |  |  |  |  |
| Underweight/healthy | 2524 | 30.84 (1.07) | 167 | 27.43 (2.78) |
| Overweight | 2677 | 34.39 (0.92) | 175 | 28.64 (3.09) |
| Obese | 2770 | 34.77 (0.86) | 295 | 43.93 (2.76) |
